# Anti‐social learning: The impact of language on mentalizing

**DOI:** 10.1111/bjop.70001

**Published:** 2025-07-01

**Authors:** Bryony Payne, Geoffrey Bird, Caroline Catmur

**Affiliations:** ^1^ Department of Psychology Institute of Psychiatry, Psychology and Neuroscience, King's College London London UK; ^2^ Department of Experimental Psychology University of Oxford Oxford UK; ^3^ Centre for Research in Autism and Education, Institute of Education University College London London UK

**Keywords:** cultural learning, language, mentalizing, out‐groups, theory of mind

## Abstract

Theories of cultural evolution suggest that humans may learn to represent others' minds through cultural practices including verbal instruction. It has been demonstrated that humans use less sophisticated mental state words when describing out‐group members compared to in‐group members, but whether this impacts on how out‐group members' minds are represented has not yet been determined. The media is one of the main ways in which information about out‐groups is shared; therefore, across three experiments we explored whether the language used in the media to describe out‐groups, specifically language about mental states, shapes how recipients represent the minds of out‐group members. All three experiments measured the extent to which participants represented out‐group members as individuals with distinctive minds. Experiment 1 compared language in a left‐leaning versus a right‐leaning UK news source. Experiment 2 tested the effect of including or omitting mental states or first‐person language, while Experiment 3 examined the impact of varying the amount of mental state language. We show that participants are more prone to take into account each out‐group member's mind when inferring their mental states when mental state language is used to introduce them. This demonstrates the clear role of cultural learning on how people think about others' minds.

## BACKGROUND

Understanding another individual involves representing the thoughts that individual has. This fundamental ability is known as theory of mind (Wellman & Liu, 2004). Recent work suggests that the first step in inferring what someone is thinking involves forming a mental representation of that individual's mind. For example, upon seeing an individual offering to help a stranger in need, one might represent that individual as being *kind*. This representation is stored in one's ‘Mind‐space’ – a multidimensional space where the dimensions reflect any characteristic of minds that allows them to be individuated, such as personality traits, like kindness, or other factors, such as intelligence (Conway et al., 2019).

Importantly, where someone represents an individual's mind in this space affects the probability of particular mental states being ascribed to them (Conway et al., 2020). For instance, if someone represents an individual as very kind, it is likely to give rise to the inference that this individual also *believes* that helping others is good for society or that they *intend* to help others when given the opportunity to do so. Representing someone's mind as lacking in kindness would give rise to very different inferences. Thus, the representation a person has of another's mind affects the inferences that person will make about the types of views, beliefs and intentions (i.e. mental states) the other is likely to possess (Conway et al., 2019, 2020). Critically, if the representation is not accurate, people are likely to make inaccurate inferences about the mental states the other holds (Conway et al., 2020). For example, people frequently make inaccurate inferences about the views and beliefs of out‐group members (Payne et al., 2024). It is therefore important to examine the processes by which people come to hold these inaccurate assumptions about the views of people outside of their own group – whether this group is defined by differences in political or religious views, geographic origin, gender or neurotype.

Theories of cultural evolution suggest that humans may learn to represent others' minds through cultural practices including verbal instruction (Heyes & Frith, 2014). This is in contrast to nativist accounts which suggest that the ability to understand others' minds is present from infancy and is not substantially changed thereafter (e.g. Carruthers, 2013). Cultural evolution accounts suggest that many aspects of cognition are learnt from others, through cultural learning practices such as reading, social learning, imitation and teaching (Heyes, 2012). Thus, it is possible we inherit ways of thinking about the minds of out‐group members from others (Heyes, 2018; Heyes & Frith, 2014).

Research has shown that children learn about the relationship between a person's mental states and their behaviours through conversations with their caregivers (Ruffman et al., 2002; Slaughter & Peterson, 2011). Yet, both adults (McClung & Reicher, 2018) and children (McLoughlin & Over, 2017) use fewer and less sophisticated mental state words when describing out‐group members compared to in‐group members and also attribute less complex emotions to them (Demoulin et al., 2004). This is thought to reflect people's tendency to represent out‐group members with less ‘humanness’ (Harris & Fiske, 2009; McLoughlin & Over, 2017). Critically, however, it has not been shown whether these descriptions impact how people represent the *minds* of in‐ and out‐group members; that is, whether such impoverished descriptions of out‐group members influence how the *recipients* of this language represent out‐group minds, and subsequently, how they infer the mental and emotional states those minds possess.

In other words, individuals appear to consider out‐group members as having less complex mental states, and this is reflected in the language used about them. However, it has not been shown whether cultural learning about out‐groups – via the medium of relatively impoverished mental state language – leads to poorer representations of the mental states of such out‐groups.

If we learn about the minds of others through cultural learning, the media is one of the main ways in which information about out‐groups is shared. That is, the media informs the public about the world, ‘particularly in those areas in which audiences do not possess direct knowledge or experience’ (Happer & Philo, 2013, p. 321). For example, people's perception and understanding of out‐groups such as immigrants, by virtue of being outside of the reader's direct knowledge, are relatively more informed by the media (Scherman et al., 2022). Therefore, it is essential to explore whether – and to what extent – the language used in the media to describe out‐groups can, through cultural learning, shape how recipients represent the *minds* of members of those out‐groups. As mentioned above, cultural evolutionary theories predict strong effects of cultural practices, including the use of particular types of language, on mental state representation, which would not be expected under nativist accounts; but this also has practical implications, since if language can impact the representation of others' minds it is critical to consider the role of language in our education systems and society.

### Overview of experiments and measures

**FIGURE 1:**
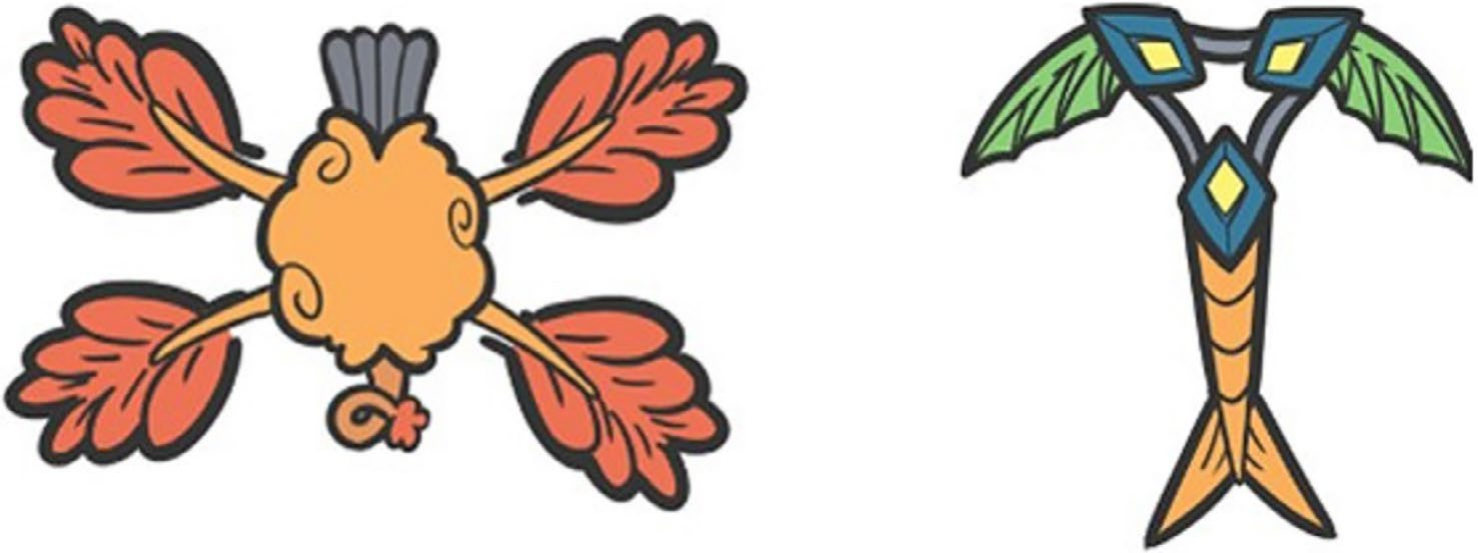
Example of images used to depict ‘Cloods’ and ‘Zyns’, two different alien species.

Immigrants are, at least geographically, an out‐group with respect to the host culture. Press coverage about immigration has been steadily increasing in the United Kingdom since 2013 (Allen, 2016) and it is one of the most divisive issues in the UK today (Cooper et al., 2021). In this study, we looked at the effect of the media language used about immigrants, as an example out‐group, on five key variables in each of three experiments. To avoid any existing representation of specific immigrant groups adding noise to the manipulations of interest, the texts were altered to be about a new alien race (‘Cloods’ or ‘Zyns’, henceforth referred to as ‘targets’; see Figure 1) rather than specific groups of immigrants.

First, we measured people's empathy towards targets after reading about them in the media. People typically exhibit higher empathy for in‐group relative to out‐group members (Tarrant et al., 2009). Thus, if the language within the media portrays immigrants as relatively more in‐ or out‐group, and if this shapes how recipients represent targets' minds, one might expect a difference in the degree of empathy the participant has for the targets.

Second, we measured the extent to which people represented targets as possessing warmth and competence. These traits are thought to be dimensions on which people stereotype other groups, and immigrants are judged to be low in both warmth and competence (Fiske, 2018). If people learn about the minds of out‐groups from others, the type of language used by the media may affect how warm and competent out‐groups are perceived to be.

The remaining variables were critical with respect to the representation of minds and mental states, which was our measure of primary theoretical interest. We wanted to assess whether the language used to describe targets could affect how prone people are to use their representation of each target's mind (i.e. which personality traits they possess) to inform their inferences about their mental states, or whether they would attribute the same beliefs to all targets (i.e. treat them as one homogenous group with the same mental states). To that end, our third variable measured the degree to which participants represented targets as possessing certain personality traits: specifically, how *rational, trusting, carefree, cautious, superstitious* and *pessimistic* they are. These traits were chosen because they are all associated with the personality trait of being paranoid (Conway et al., 2020). Paranoia was chosen because of its relevance to mental state inference in certain situations (see below). The traits *rational, carefree* and *trusting* are each negatively correlated with being paranoid, while the traits *superstitious, pessimistic* and *cautious* are each positively correlated with paranoia. By knowing how the participant believes these traits are related to paranoia, one can calculate the degree to which they estimate targets are paranoid (the fourth variable) without directly asking them (and thus avoiding demand characteristics; Conway et al., 2020). Finally, using these variables, we examined the extent to which participants used their representation of how paranoid a particular target is to infer their mental state in a situation in which paranoia is relevant to mental state inference (the fifth variable), instead of just attributing a stereotypical belief to all targets. This final analysis was of key theoretical importance regarding the cultural evolution of the ability to infer others' mental states, as it measures the extent to which each target is considered as an individual with their own mind and resulting mental states.

Experiment 1 compared the impact of language used by a left‐leaning UK news source (The Guardian) versus a right‐leaning UK news source (The Daily Mail) on these variables. While both sources might consider immigrants to be out‐group members with respect to their readership, the different political stance of each source should result in the left‐leaning paper (The Guardian) using language that treats immigrants as relatively more in‐group than the right‐leaning paper (The Daily Mail; see Berry et al., 2016). This initial experiment used relatively naturalistic and uncontrolled stimuli to explore the effect of media language on the representation of targets' minds, meaning that any effects could be due to a number of factors. The subsequent experiments followed up Experiment 1 using more controlled stimuli, focusing on two of the possible linguistic differences which could be driving the observed effects. Experiment 2 tested the effect of explicitly including or omitting mental states or first‐person language on the representation of targets, while Experiment 3 examined the impact of varying the amount of mental state language (rather than just examining its presence vs. absence).

To summarize, we investigated how the language used by the media in reference to targets (i.e. immigrants) influences: (1) the level of empathy people feel towards out‐group members; (2) the extent to which people represent out‐group members as possessing certain personality traits, including warmth and competence; and (3) people's propensity to use their representation of out‐group minds when making inferences about their mental states.

## EXPERIMENT 1

Experiment 1 introduced participants to two alien races, with one alien race introduced by articles taken from the politically left‐leaning news source, and the other from the politically right‐leaning news source. Participants' empathy towards the targets, their personality attributions and their propensity to consider individual targets' minds when inferring their mental states were subsequently measured to assess whether these factors were impacted by the language used when the out‐group targets were discussed in the two news sources.

### Methods

#### Transparency and openness

We report how we determined our sample size, all data exclusions, all manipulations and all measures in the study. All experiments were pre‐registered, including the plans for sampling, exclusions and analyses. All pre‐registrations and data are available on the OSF at https://osf.io/6tvsd/?view_only=b4e19e9ff5e1483581abc534aea95874.

#### Participants

We recruited a final sample of 128 participants (mean age = 47.71 years, SD = 15.06 years, age range = 18–104,^1^ 64 female and 64 male). All participants were recruited online via Prolific (www.prolific.co.uk), self‐reported as neurotypical, with English as their primary language and as a UK national. We made the decision to recruit solely from the United Kingdom because the stimuli were generated from UK news sources, and we wanted to select participants who would in principle have some familiarity with these sources and the types of news on which they report (although participants were not informed about the sources of the articles). Participants reported no significant visual impairments, mild cognitive impairments or dementia. Finally, participants had to have a current approval rating on Prolific of over 90% for data quality reasons. None had taken part in any pilot studies associated with this project and, upon completion of the study, were paid for their participation. Ethical approval was obtained from the local Health Faculties Research Ethics Subcommittee, and informed consent was obtained from all participants prior to testing.

This sample size was determined by an a priori G*Power calculation for the fifth analysis (Analysis: v) aiming to obtain .80 power to detect a medium effect size of .25 at the standard .05 alpha error probability while modelling a two‐way interaction between news source (left‐leaning vs. right‐leaning) and target paranoia scores on mental state (false belief) inference.

#### Stimulus development

##### Selecting articles

To minimize researcher bias in the selection of articles about immigration, a systematic approach to accumulate news articles from both sources was applied. An advanced search was conducted on The Daily Mail (right‐leaning) and The Guardian (left‐leaning) sources, using the terms: “*Immigrant*” OR “*Migrant*”, OR “*Asylum Seeker*” OR “*Refugee*”, between the time frame 13th– 20th of October 2023. Any articles that were formatted as a letter, video blog, opinion piece or live webpage were excluded. This yielded a total of 78 articles (right‐leaning = 40, left‐leaning = 38). Thereafter, any articles that covered the same world event were removed, to avoid participants confusing the two sources. This left 54 articles (right‐leaning = 35, left‐leaning = 19), from which 5 were randomly selected from each source for use as stimuli in Experiment 1.

##### Adapting the articles

Each article was shortened using the first 350 words, to the end of the nearest sentence. Further, several key terms relating to specific immigrant groups were altered or removed to make alien species called ‘Cloods’ or ‘Zyns’ the immigrant groups. Specifically, we (a) replaced the terms: ‘asylum seeker’, ‘immigrant’, ‘migrant’ and ‘refugees’ with either ‘Clood’ or ‘Zyn’; (b) replaced any proper nouns, that is names of real humans, with fictional names such as ‘Cloodvale’ or ‘Zyndell’; (c) replaced the names of places, for example Glasgow, with fictional names such as ‘Hidburn’ or ‘Adrelas’; (d) anonymized key roles (e.g. Prime Minister), organizations (e.g. Home Office) and political leanings (e.g. Republican views) with less recognizable names such as ‘Governmental leader’, ‘Clood processing department’ and ‘anti‐Clood attitudes’, respectively; (f) removed references to real‐world events such as ‘COVID‐19’ and; finally, (g) replaced any terms that are highly associated with immigration, for example ‘seeking asylum’ and ‘applying for a permit to stay’.

Importantly, the word ‘Clood’ was assigned to the right‐leaning source and ‘Zyn’ to the left‐leaning source or vice versa (counterbalanced across participants). Fictional names of targets were counterbalanced across participants to ensure that the target's name could not be responsible for any observed effect of news source. Similarly, the visual stimulus used to illustrate the target was also counterbalanced across participants.

#### Procedure

##### Task 1

All participants were tested online using Gorilla (gorilla.sc, Anwyl‐Irvine et al., 2019). Across ten trials, participants were presented with 10 text articles (one per trial); 5 from the left‐leaning source and 5 from the right‐leaning source. The articles were blocked together, such that participants either read all 5 left‐leaning articles first or all 5 right‐leaning articles first. However, the order in which articles were presented in each block was fully randomized, and participants were not given any information about the news sources from which the articles were extracted.

On each trial, participants were introduced to a particular target with a visual stimulus (see Figure 1) and a text description, for example ‘This is Cloodley, they are a Clood’. Both the name and the pronoun were gender‐neutral. No other information was given. Participants were then asked to read an article that had been, for example ‘written about Cloods like Cloodley’. The article was then presented on‐screen to the participant. After 30 seconds, a multiple‐choice, attention‐check question appeared on‐screen, requiring a verbatim answer from the text. Any participant who gave more than one incorrect answer was not allowed to continue. After the attention‐check question, participants were asked 11 questions on each trial, (i.e. 11 questions about an individual target) based on the article they had read, which remained on‐screen.

Specifically, participants were asked to state ‘how bad they felt for the target’ and ‘how inclined they were to help the target’ after reading the article. These questions were measured on a scale of 0–100, where 0 denoted ‘not at all’ and ‘100’ denoted ‘Very’ and examined participants' empathy towards targets (see Analysis: (i)). On the same scale, participants were also asked to state the degree to which they thought the target was *warm* and *competent* (Analysis: (ii)) and, moreover, how *rational, trusting, carefree, cautious, pessimistic* and *superstitious* they were (Analysis: (iii)). Note that the latter six ‘source traits’ were also used to generate a predicted paranoia score, indexing the target's paranoia (used in analyses (iv) and (v)).

All questions were presented in randomized order apart from the final question below, which measured the participants' propensity to attribute a false belief to the target (Analysis: (v)). For instance, participants were shown a scenario such as:

Cloodley puts their house keys on the kitchen counter in the communal kitchen and then leaves the room to pack their bag for the day. Meanwhile, a janitor working in the building picks up the keys and, thinking them lost, moves them to the lost and found box. When Cloodley returns to get the keys, where are they likely to search for them?

After reading the scenario, participants were asked to state the probability that a target would hold an expectation that corresponds to the false belief (the kitchen counter in the story above) compared to the true belief (the lost and found box in the story above). It has previously been shown that inference of mental states in this task is sensitive to the participant's representation of the target's level of paranoia (Conway et al., 2020): if the participant represents the target (Cloodley) to be relatively more paranoid, they should be more likely to infer that the target will also expect their keys to have been moved by someone else (i.e. are less likely to hold a false belief), compared to a target that is not considered paranoid at all.

For this variable, responses to the scenarios were measured via a sliding scale ranging from 0 to 100 (though participants could not see the numbers on the scale), such that a rating of 50 indicated that neither expectation was more likely. The end of the scale labelled with the ‘false’ belief was counterbalanced across trials, thus the data were re‐coded such that ratings closer to 0 indicated greater probability of the target holding the true belief, and ratings closer to 100 indicated greater probability of the target holding a false belief. Each scenario included a second character that was an anonymous human, rather than another Clood/Zyn target. The assignment of the ten scenarios to particular trials (i.e. to particular targets and articles) was randomized but fixed across participants.

##### Task 2

Participants were asked to state the likelihood that ‘someone who was considered “paranoid” would also be considered to be “X”’, where ‘X’ was carefree, rational, trusting, cautious, pessimistic and superstitious. These questions were each measured on a scale ranging from −100 to 100, where a higher score indicated a stronger, positive correlation between paranoia and the trait, an increasingly negative score indicated a stronger negative correlation between paranoia and the trait and ‘0’ represented no correlation between paranoia and the trait. People completed these questions about Cloods and Zyns, in turn, in counterbalanced order. These trait‐paranoia association ratings were used, alongside the personality trait ratings, to generate a ‘mean predicted relative paranoia score’. Specifically, for each participant, on each trial (i.e. for each target), we divided the score they gave a target on a source trait (e.g. rational) by 100 and multiplied it by the degree to which the participant thought that source trait was associated with paranoia (trait‐paranoia association scores were also divided by 100). These scores – one for each trait – were then summed across traits, to give one final mean predicted target paranoia score per participant, per trial. This ‘target paranoia’ score therefore represents how paranoid a participant *should* think a target is, if the score they gave that target on a particular source trait (e.g. rational) enables the participant to know how paranoid they are, based on the participant's estimated correlation between that source trait and paranoia. This measure is used in Analyses: (iv) and (v).

#### Exclusion criteria

Two participants who failed more than one attention check were removed and their data replaced, as per the pre‐registered criteria, to reach a final sample size of 128 participants.

### Results

For all analyses, trial‐wise outliers 1.5 times the interquartile range above the third or below the first quartile were removed. For all analyses using mixed‐effect models, we established statistical significance via likelihood ratio tests by comparing a full model to a reduced model without the effect of interest. We repeated this process iteratively, testing each fixed effect by removing it from an alternate reduced model to assess its contribution. As such, we report the significance of model fit changes via χ^2^ tests and their associated *p*‐values. Thus, the reported *p*‐values represent the significance of the difference between the models. In all models reported in the paper, fixed effects were coded using the default treatment coding implemented by *lmer* (i.e. dummy coding with a reference level), unless explicitly stated otherwise. Specifics of model structure varied by analysis but generally adhered to: DV ~ News Source * [other variable, where present] + (1 | Participant) in Experiment 1 (see subsequent results sections for model structure for Experiments 2 and 3 and analysis script on OSF for full details). Additionally, where relevant, we report pairwise comparisons using *emmeans* (Lenth, 2016), Bonferroni corrected for multiple comparisons.

**FIGURE 2:**
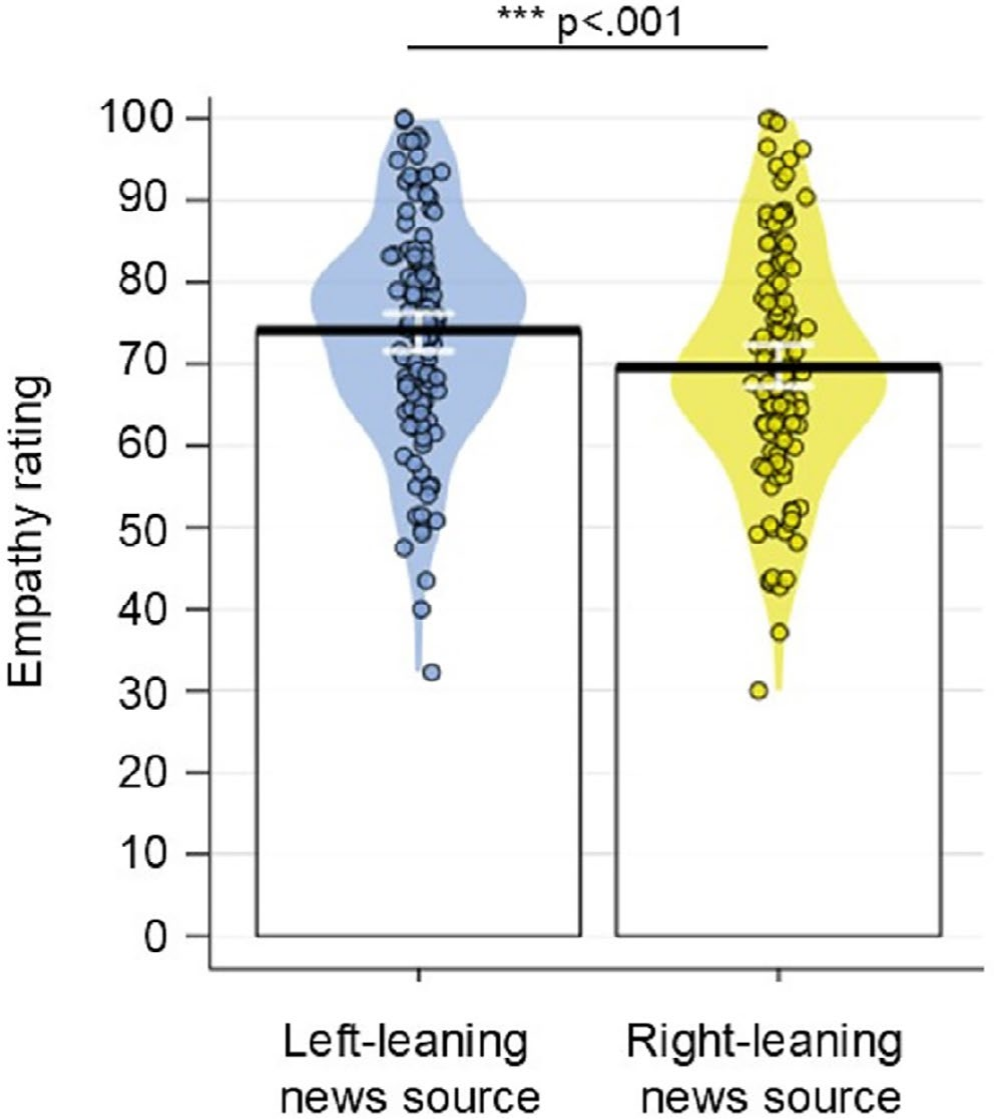
Mean empathy rating for targets introduced by right‐ vs left‐leaning news sources in Experiment 1. Coloured segments show smoothed density curves for the full data distribution, while individual dots indicate mean percentage per participant. Error bars show 95% CIs. Horizontal bars show post‐hoc comparisons with asterisks denoting significance as determined via likelihood ratio tests (see Results).

**FIGURE 3:**
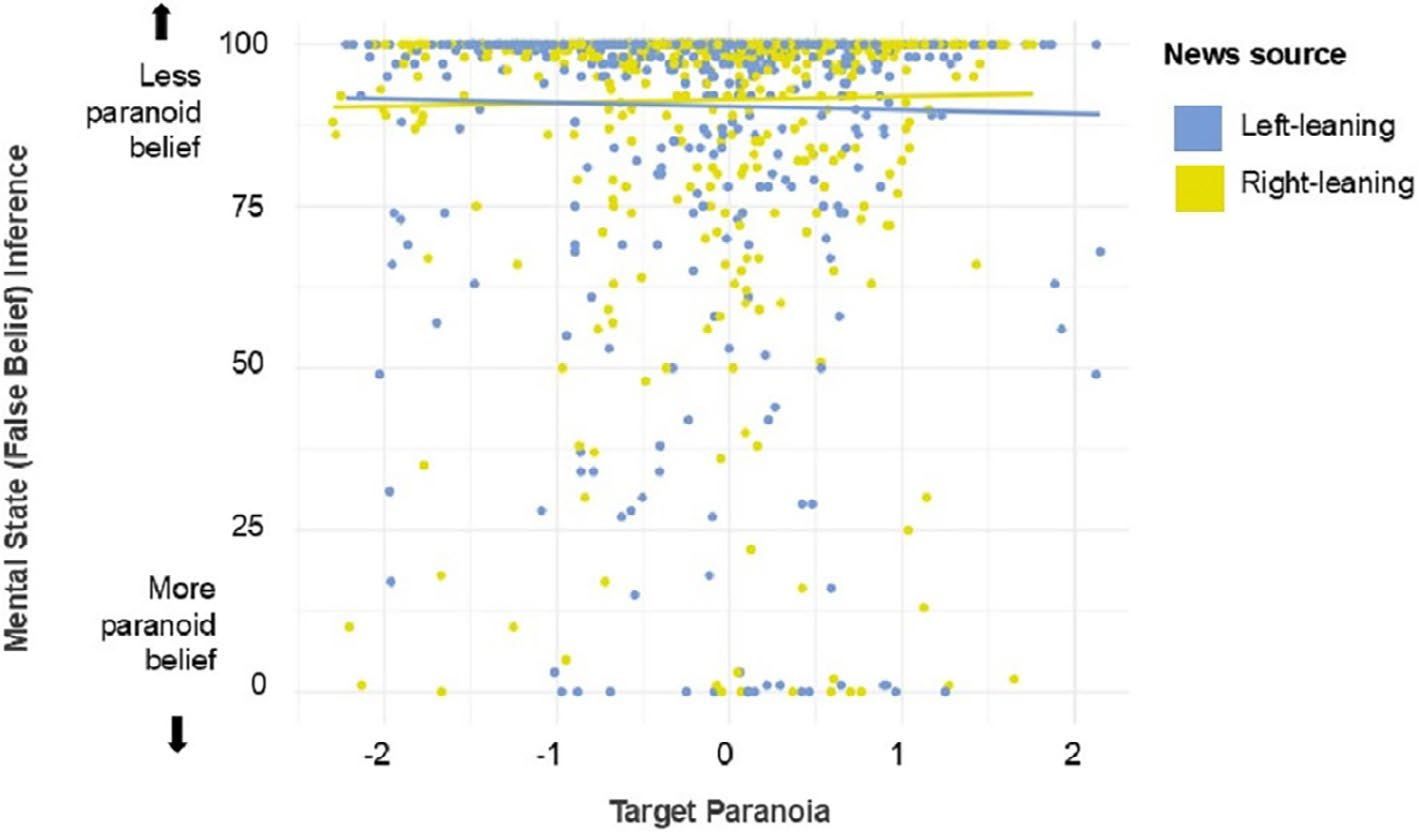
Mental state (false belief) inferences as a function of target paranoia in Experiment 1. High values indicate participants inferred the target to hold a less paranoid belief, lower values indicate inference of a more paranoid belief. When targets were introduced by a right‐leaning news source, participants failed to individuate targets. However, when targets were introduced by a left‐leaning news source, participants took into account target individuals' levels of paranoia more when inferring their mental states. Solid lines represent the linear regression for each news source while individual dots indicate mean scores per participant.

Descriptive statistics for participants' ratings of the six source traits as well as warmth and competence are reported in Table 1. Empathy scores are shown in Figure 2, while scores indexing target paranoia and mental state (false belief) inference are shown in Figure 3.

**TABLE 1:** Mean (SD) ratings of each personality trait by news source in Experiment 1.

News source	Competent	Warm	Carefree	Cautious	Pessimistic	Rational	Superstitious	Trusting
Right‐leaning	60.59 (12.88)	54.04 (12.72)	25.68 (14.91)	64.77 (16.33)	67.13 (15.42)	58.71 (15.27)	38.91 (18.45)	38.30 (15.38)
Left‐leaning	68.34 (12.09)	63.08 (13.06)	26.76 (13.59)	66.61 (13.75)	64.19 (12.49)	66.47 (14.19)	37.83 (19.46)	44.23 (16.02)

#### Empathy

The first pre‐registered analysis assessed whether news source (left‐leaning vs. right‐leaning) influenced participants' empathy towards targets. Mixed‐effect models, which included the fixed effect of news source (left‐ vs. right‐leaning) and ‘participant’ as a random intercept, showed that the main effect of news source was significant (χ^2^(1) = 73.951, *p* < .0001). Specifically, participants reported feeling more empathetic towards targets who had been introduced by the left‐leaning articles, relative to targets introduced by the right‐leaning articles (see Figure 2).

#### Warmth and competence

The second pre‐registered analysis assessed whether news source influenced participants' perception of targets as being warm or competent (analysed together to allow any interaction to emerge, as per Fiske (2018)). Mixed‐effect models, which included the fixed effects of news source and dimension (warmth vs. competence), as well as the interaction between them, and ‘participant’ as a random intercept, determined that the interaction between news source and dimension was not significant (χ^2^(1) = 1.836, *p* = .176). Rather, there was a main effect of news source (χ^2^(1) = 242.58, *p* < .0001), such that participants perceived targets introduced by the left‐leaning articles to be both more warm *and* more competent than targets introduced by the right‐leaning articles. Lastly, there was a main effect of dimension (χ^2^(1) = 121.81, *p* < .0001) such that, across both left‐ and right‐leaning articles, participants perceived targets to be less warm than competent.

#### Personality traits related to paranoia

The third pre‐registered analysis assessed whether news source influenced participants' representation of the targets' personality traits. For each trait, we ran mixed‐effect models which included the fixed effect of news source and ‘participant’ as a random intercept. Participants perceived targets to be more rational (χ^2^(1) = 83.643, *p* < .0001), more trusting (χ^2^(1) = 34.545, *p* < .0001) and less pessimistic (χ^2^(1) = 8.9944, *p* < .003) when they were introduced via the left‐leaning compared to the right‐leaning articles. The other traits did not significantly differ between news sources.

#### Predicted paranoia

The fourth pre‐registered analysis assessed whether news source influenced participants' predictions of how paranoid a target is. A paired samples *t*‐test was non‐significant (*t*(126) = 1.4873, *p* = .139), suggesting that participants did not predict targets to be differently paranoid when introduced by left‐leaning (mean = −.15, SD = .80) vs. right‐leaning (mean = −.07, SD = .80) articles.

#### Propensity to use trait information to inform inferences about mental states

In the fifth and final pre‐registered analysis, we analysed whether news source influenced participants' propensity to use their representation of targets' minds (i.e. which personality traits they possess) to inform their inferences about a target's mental states. This analysis used target paranoia as a predictor variable, along with news source and the interaction between them. The dependent variable was the mental state (i.e. false belief) inference. The logic of this analysis is as follows: in a standard false belief scenario, paranoid individuals will think that the false belief location is less likely than non‐paranoid individuals. For example, paranoid individuals are more likely to believe that someone might have taken their object (see Conway et al. (2020) for an empirical demonstration that participants do indeed predict that paranoid characters believe their object is more likely to be in the true rather than false belief location). If cultural learning via language results in a tendency to treat all out‐group members as the same (i.e. without individuation), then paranoia scores for individual targets would explain less variance in the inferences participants make about targets' mental states than if out‐group targets were individuated such that mental states were inferred based on the participant's estimate of the target's degree of paranoia. This analysis therefore assessed whether a target's paranoia influenced participants' inference about the target's mental state (i.e. whether they hold a false belief), and whether this influence differed according to how the target has been introduced – via left‐ vs. right‐leaning articles.

Mixed‐effect models showed that the interaction between target paranoia and news source on mental state inference was significant (χ^2^(1) = 3.87, *p* = .049), such that the influence of target paranoia on mental state inference was stronger for targets introduced by left‐leaning relative to right‐leaning articles (see Figure 3). This suggests that participants were more prone to use their representation of a target's mind to make inferences about their mental states – specifically, judging that targets higher in paranoia would be less likely to hold a false belief – when they had been introduced by the left‐leaning articles, compared to the right‐leaning news articles. The effects of news source and target paranoia were not significant.

### Discussion

Overall, Experiment 1 demonstrated that some aspect of the way in which immigrants are presented in left‐ vs. right‐leaning news sources influences (a) the degree of empathy people have towards out‐group members, (b) the personality traits people perceive them to have and (c) how prone they are to consider out‐group members as individuals with their own minds when inferring their mental states. This is consistent with cultural evolutionary perspectives on mental state representation (e.g. Heyes & Frith, 2014) which predict that cultural practices including verbal instruction have a strong influence on how people represent others' minds. However, the media articles used in Experiment 1 vary in a number of ways. Experiment 2 therefore provides a more systematic test of the effects of specific linguistic factors on how others' minds are represented.

## EXPERIMENT 2

Exploratory textual features analyses of the language used in the news sources (see Supplementary Material) suggested that from a linguistic perspective, some of the effects may have been driven by one or both of the presence of mental state language and the use of first‐person language. Experiment 2 provided a direct test of the effect of these linguistic factors on attitudes to out‐group members (and therefore the role of cultural learning on how we think about others' minds), through direct manipulation of the language in the articles from the left‐leaning news source, such that it either did or did not contain mental state and first‐person language.

### Method

#### Participants

In Experiment 2, we recruited 256 new participants (mean age = 48.09 years, SD = 14.13 years, age range = 20–79, 128 female and 128 male), recruited with the same eligibility criteria via Prolific (prolific.co.uk) and tested via Gorilla (gorilla.sc).

#### Stimulus development

Only articles from the left‐leaning news source (The Guardian) were used as the right‐leaning news source (The Daily Mail) included too few examples of either mental states or first‐person language to be able to examine any effect of their removal. Relatedly, while four of the original five left‐leaning articles were reemployed in Experiment 2, one was dropped because it contained no first‐person language. Two new articles were chosen from the left‐leaning news source to bring the total stimulus set up to six articles. The two additional articles were chosen from the pool of articles originally selected at the beginning of Experiment 1. Of the articles that were available from that pool, two articles with the highest amount of mental state language and first‐person language were chosen. All the articles were adapted to be about ‘Cloods’ (i.e. not Zyns).

The six chosen articles were then further adapted to create 4 new sets: (1) the original articles; (2) with mental states removed; (3) with first‐person language removed (i.e. pronouns referring to ‘I’ ‘me’ ‘my’ ‘mine’ ‘we’ ‘us’ ‘our’ and ‘ours’ were changed to third person pronouns); or (4) with both mental states and first‐person language removed.

#### Design

In Experiment 2, we employed a between‐subjects design such that participants were randomly assigned one of the four stimulus sets. As such, there were two between‐subjects factors: mental state language (present vs. absent) and first‐person language (present vs. absent).

#### Procedure

The procedure was identical to Experiment 1 except for the following. In Task 1, only six of the original ten false belief scenarios were utilized, one per trial. Which six were used was randomized, and the assignment of scenario to article was again randomized but fixed across participants. Second, in Task 2, participants were only asked about the trait‐paranoia association ratings for Cloods, as Zyns did not feature in the Experiment. The measured variables were identical to Experiment 1.

#### Exclusion criteria

Three participants were excluded and replaced based on pre‐registered exclusion criteria. Specifically, one participant was excluded because they responded to more than 20% of perceived traits questions in less than 1000 ms and two participants were excluded because they failed more than one attention check.

### Results

Descriptive statistics for participants' ratings of targets' personality traits and empathy towards targets are reported in Table 2.

**TABLE 2:** Mean (SD) ratings of empathy and each personality trait by condition in Experiment 2.

Mental states	First‐person language	Empathy	Competent	Warm	Carefree	Cautious	Pessimistic	Rational	Superstitious	Trusting
Present	Present	62.94 (21.87)	66.34 (12.50)	62.83 (13.17)	37.49 (14.84)	61.38 (13.69)	57.88 (14.02)	64.17 (11.54)	35.75 (18.69)	51.62 (14.71)
	Absent	63.10 (21.06)	65.44 (14.31)	62.49 (12.69)	33.86 (16.37)	61.16 (14.76)	57.81 (16.53)	64.80 (12.49)	39.93 (18.08)	48.16 (17.19)
Absent	Present	66.92 (19.61)	67.98 (11.69)	60.72 (15.28)	32.96 (16.56)	61.23 (16.51)	60.40 (14.98)	64.83 (14.48)	36.25 (19.95)	46.26 (17.03)
	Absent	62.86 (22.16)	64.73 (13.42)	59.05 (13.08)	29.45 (13.35)	62.38 (14.71)	59.80 (13.63)	63.97 (12.51)	39.74 (17.76)	44.20 (14.22)

For all analyses, as all articles were from a single news source, ‘article’ could be included as a random effect to account for any item‐specific variability without overlapping with other predictors (this was not possible in Experiment 1 because, by design, article was confounded with news source). Model structure therefore adhered to: DV ~ Mental States * [other variable, where present] + (1 | Participant) + (1 | Article), or DV ~ First Person Language * [other variable, where present] + (1 | Participant) + (1 | Article).

#### Empathy

Mixed‐effect models assessed the main effects of mental state language (present vs. absent) and of first‐person language (present vs. absent) – as well as any interaction between them – on participants' empathy ratings, including ‘Participant’ and ‘Article’ as random intercepts. The main effects of mental states (χ^2^(1) = 0.49, *p* = .485) and of first‐person language (χ^2^(1) = 0.56, *p* = .455) were both non‐significant, as was the interaction between the two (χ^2^(1) = 0.64, *p* = .425).

#### Warmth and competence

Mixed‐effect models assessed the main effects of mental state language, first‐person language and dimension (warmth vs. competence) – as well as any interaction between them – on participants' ratings of warmth and competence of the targets, including ‘Participant’ and ‘Article’ as random intercepts. The two‐way interaction between mental states and dimension was significant (χ^2^(1) = 9.483, *p* = .002). Pairwise comparisons show that the ratings of competence did not increase in the presence of mental states (*z* = 0.272, *p* = .786) but there was a statistical trend towards ratings of higher warmth (*z* = 1.786, *p* = .074) in the presence of mental states. The two‐way interaction between first‐person language and dimension was non‐significant (χ^2^(1) = 1.00, *p* = 0.316), as were the main effects of mental states (χ^2^(1) = 0.64, *p* = 0.422) and first‐person language (χ^2^(1) = 1.11, *p* = 0.292). There was, however, a main effect of dimension (χ^2^(1) = 84.613, *p* < .001), reflecting the finding that participants rated targets to be more competent than warm.

#### Personality traits related to paranoia

Mixed‐effect models assessed the main effects of mental state language and first‐person language on participants' ratings of each personality trait, including ‘Participant’ and ‘Article’ as random intercepts. The analysis showed that participants perceived targets to be both more trusting (χ^2^(1) = 5.55, *p* = .019) and more carefree (χ^2^(1) = 5.46, *p* = .02) when those targets had been introduced with articles that included mental states, in comparison to articles that did not. The other traits were unaffected by the presence of mental states (all χ^2^(1) < 1.5, all *p* > .2). The presence of first‐person language did not affect the representation of any traits (all χ^2^(1) < 3.5, all *p* > .06).

#### Predicted paranoia

The *t*‐test assessing the effect of the presence of mental states was significant (*t*(1475.7) = 3.159, *p* = .002, present: mean = −.24, SD = .85, absent: mean = −.36, SD = .77), such that participants represented targets to be more paranoid when the language included mental states, compared to when it did not. The *t*‐test assessing the presence of first‐person language was not significant (*t*(1471.1) = 0.836, *p* = .403; present: mean = −.32, SD = .75, absent: mean = −.28, SD = .86).

#### Propensity to use trait information to inform inferences about mental states

Mixed‐effect models assessed the main effects of the presence of mental state language, first‐person language and target paranoia – as well as the interaction between them – on participants' mental state inference. None of the main effects nor interaction effects were significant (all χ^2^(1) < 1.8, all *p* > .2).

### Discussion

The results of Experiment 2 suggest that the presence of mental states in language about out‐groups causes members of those out‐groups to be judged as having higher levels of certain personality traits (more trusting, more carefree) and a trend towards being more warm; but did not suggest that it makes people more prone to use those representations of them when inferring their mental states. This latter result was similar for the presence of first‐person language.

Given the small effect size present in Experiment 1, it may be that the content of the article (i.e. its narrative) reduced the size of the effect of the presence of mental states by introducing noise. While ‘article’ was included as a fixed effect in all analyses, it was not factorially randomized, so we could not fully control for the effect of its content. Moreover, even in the instances where mental states were present in the language, the articles varied in the number of mental states they contained (*M* = 15.8, SD = 5.8) such that, across articles, the number may not have been sufficient to detect an effect of their presence compared to their absence.

As such, in Experiment 3, we aimed to parameterize the presence of mental states to test if there is a ‘dosage‐dependent’ effect of mental states, such that people only become more prone to consider out‐group minds when making mental state inferences about them once the language used to introduce them contains a certain number of mental states. In Experiment 3, therefore, all articles used in Experiment 2 were manipulated such that they either contained no mental states, a medium amount of mental states (12 per article), or a high amount of mental states (24).

In Experiment 2, neither first‐person nor mental state language affected how empathetic participants were towards targets. However, our empathy measure was made up of two questions, one relating to affect sharing and the second to empathic concern. It is possible that the change to using only the left‐leaning news source impacted these questions differentially, and thus, for Experiment 3, we pre‐registered that we would analyse the two empathy questions separately, allowing any interaction with the empathy question to emerge.

## EXPERIMENT 3

### Methods

#### Participants

Two hundred new participants (mean age = 43.11 years, SD = 14.82 years), age range = 18–80 (100 female and 100 male) were recruited with the same eligibility criteria via Prolific (prolific.co.uk) and tested via Gorilla (gorilla.sc).

#### Stimulus development

The six Guardian articles used in Experiment 2 were used again in Experiment 3. However, each of the articles was manipulated to contain no mental states, a medium number (12) or a high number (24). The ‘high’ amount was determined by the maximum amount of mental state words included in any of the articles in Experiment 2. The remaining articles were subsequently altered to include a higher number of mental states than they originally did. Thereafter, alternate mental states were systematically removed until each article only contained 12 states (constituting the medium level). Lastly, all mental states were removed to constitute the ‘no mental states’ level.

Given that, throughout these studies, cognitive mental states have not been separated from emotional mental states, and to control for the potentially differential effects of either, the number of cognitive vs. emotional states was controlled such that each ‘high’ version of the articles contained 18 cognitive states and 6 emotional states, while each ‘medium’ version contained 9 cognitive states and 3 emotional states.

#### Design

Experiment 3 employed a within‐subjects design with 1 factor: mental state language (with 3 levels: none, medium, high). Within the 3 different conditions, participants completed 2 trials (6 trials in total). The article that was assigned to each of the different conditions was fully randomized and counterbalanced across participants. Assignment of the different false belief scenarios was fully randomized across articles.

#### Procedure

The procedure in Experiment 3 was identical to Experiment 2.

#### Exclusion criteria

One participant who failed more than one attention check was removed from the study and their data replaced, as per the pre‐registered criteria.

### Results

**FIGURE 4:**
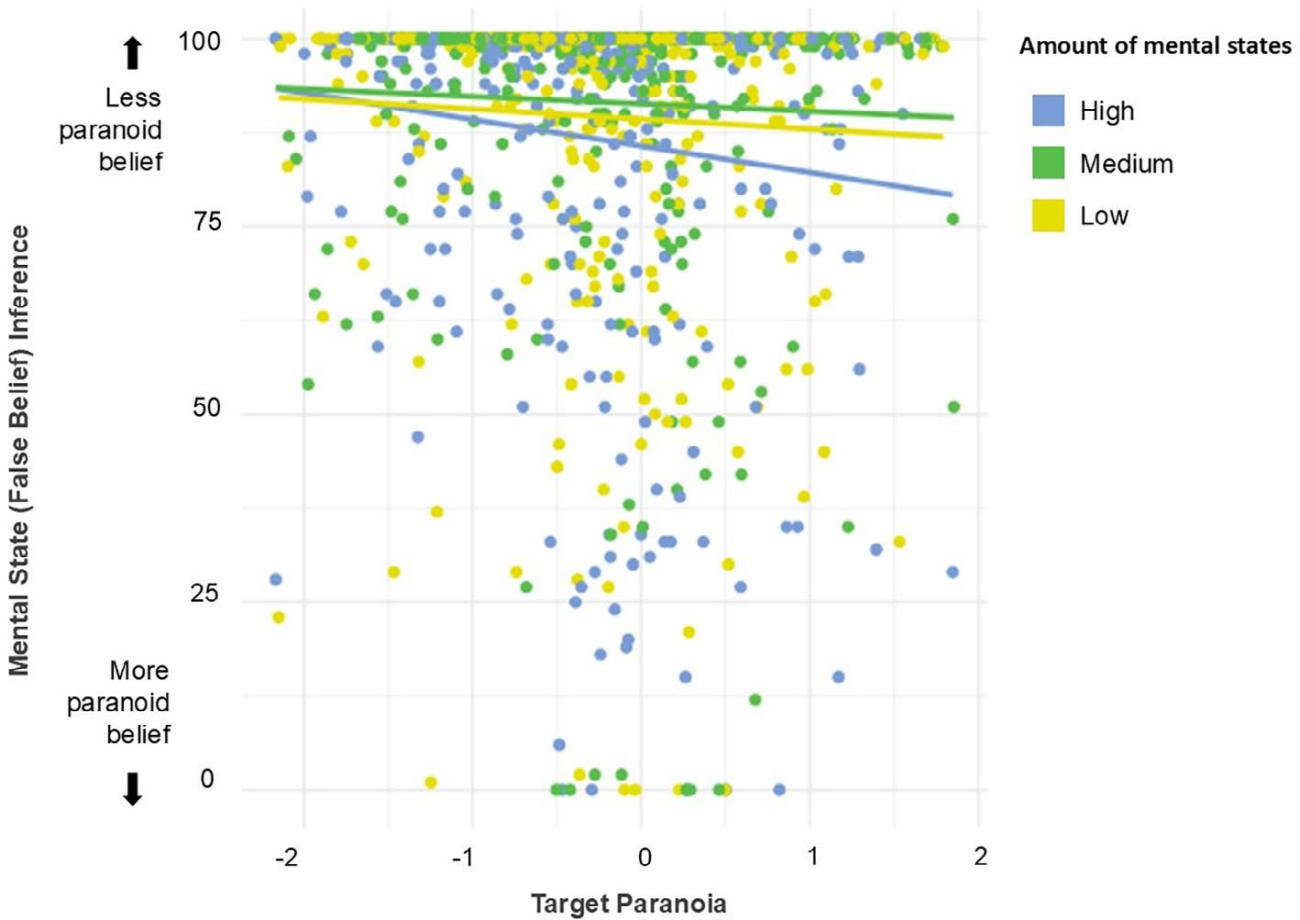
Mental state inferences as a function of target paranoia in Experiment 3. High values indicate participants inferred the target to hold a less paranoid belief, lower values indicate inference of a more paranoid belief. When targets were introduced by language that contained a high number of mental states, they took into account target individuals' levels of paranoia more when inferring their mental states. Solid lines represent the linear regression for each level of mental state language, while individual dots indicate mean rating per participant.

Descriptive statistics for participants' empathy towards targets in Experiment 3 are reported in Table 3, while participants ratings of targets' personality traits are reported in Table 4. Scores indexing target paranoia and participant's mental state (false belief) inference are plotted in Figure 4.

**TABLE 3:** Mean (SD) ratings of empathy by amount of mental states in Experiment 3.

Mental state	Empathy: Affect sharing	Empathy: Empathic concern
High	71.86 (17.88)	69.38 (17.53)
Medium	72.84 (18.93)	69.39 (18.28)
None	72.25 (17.41)	68.87 (18.57)

**TABLE 4:** Mean (SD) ratings of personality traits by amount of mental states in Experiment 3.

Mental states	Competent	Warm	Carefree	Cautious	Pessimistic	Rational	Superstitious	Trusting
High	68.14 (15.96)	63.08 (14.96)	33.75 (18.39)	61.59 (20.24)	58.76 (18.23)	63.43 (16.42)	37.45 (18.36)	50.99 (18.93)
Medium	67.95 (13.98)	61.48 (14.91)	31.36 (19.52)	62.05 (19.13)	61.48 (18.15)	65.55 (16.40)	37.47 (19.25)	47.19 (20.16)
None	67.53 (14.39)	62.86 (14.99)	31.70 (19.49)	59.68 (19.69)	59.88 (19.09)	65.73 (17.01)	37.46 (19.72)	46.65 (19.54)

For all analyses, we did not include ‘article’ as a random effect because article‐specific variance was evenly distributed across conditions (since assignment of each article to each mental state language condition was fully counterbalanced across participants). Model structure therefore adhered to: DV ~ Mental State Language * [other variable, where present] + (1 | Participant). Mental state language (three levels: high, medium, none) was coded using the default treatment coding in lmer, with ‘High’ as the reference level.

#### Empathy

Mixed‐effect models assessed the main effect of mental state language (none, medium, high) and empathy question (affect sharing vs. empathic concern) – as well as any interaction between them – on participants' empathy rating for targets, including ‘Participant’ as a random intercept. Neither the interaction between the amount of mental states and the empathy question (χ^2^(2) = 0.540, *p* = .763) nor the main effect of mental states (χ^2^(2) = 0.322, *p* = .851) were significant. There was a main effect of the empathy question asked (χ^2^(1) = 23.383, *p* < .0001), such that participants gave higher ratings when asked how bad they felt for the target (affect sharing), compared to how inclined they were to help (empathic concern).

#### Warmth and competence

Mixed‐effect models assessed the main effect of mental state language and dimension (warmth vs. competence) – as well as any interaction between them – on participants' ratings of the targets, including ‘Participant’ as a random intercept. The two‐way interaction between mental states and dimension was not significant (χ^2^(2) = 2.577, *p* = .276), and neither was the main effect of mental states (χ^2^(2) = 1.344, *p* = .511). There was a main effect of dimension (χ^2^(1) = 77.385, *p* < .001), such that targets were perceived to be more competent than warm.

#### Personality traits related to paranoia

Mixed‐effect models assessed the main effect of mental state language on ratings of each of the six personality traits, including ‘Participant’ as a random intercept. There was a significant main effect of mental states on how trusting participants perceived targets to be (χ^2^(2) = 12.171, *p* = .002). Pairwise comparisons via *emmeans* showed that when targets were introduced with language using a high number of mental states, they were perceived as being significantly more trusting than when the language used a medium number of mental states (*t*(1002) = 2.806, *p* = .015) or no mental states (*t*(1002) = 3.208, *p* = .004). There was no difference in perceived trustingness for medium relative to no mental states (*t*(1002) = 0.402, *p* = .999). The other traits were unaffected by the amount of mental states (all χ^2^(2) < 5.6, all *p* > .06).

#### Predicted paranoia

Mixed‐effect models determined whether participants predicted targets to have significantly different levels of paranoia, based on the number of mental states used. The main effect of mental states did not reach statistical significance (χ^2^(2) = 5.652, *p* = .059; none: mean = −.21, SD = .76; medium: mean = −.19, SD = .78; high: mean = −.24, SD = .76).

#### Propensity to use trait information to inform inferences about mental states

Mixed‐effects models assessed the main effects of mental state language and target paranoia – as well as the interaction between them – on participants' mental state inference. The models included ‘Participant’ as a random intercept. The two‐way interaction between mental state language and target paranoia was not significant (χ^2^(2) = 2.194, *p* = .334). We conducted a follow‐up contrast to directly test our primary theoretical comparison – between ‘High’ and ‘None’ – while excluding the ‘Medium’ condition. This was implemented via the contrast function on the marginal means using weights (1, 0, −1) (see OSF script for details). The contrast revealed a significant effect (*t*(940) = 2.446, *p* = .015; see Figure 4), indicating that participants were more prone to use their representation of targets' minds when the language used to introduce them contained a high number of mental states, compared to when it contained no mental states.

Further, there was a main effect of the amount of mental states on participants' mental state inference (χ^2^(2) = 16.014, *p* = .0003), such that when there was a higher number of mental states, participants were less likely to think that the target held a false belief compared to when there was a medium amount (*t*(939) = 3.985, *p* = .0002) or none (*t*(938) = 2.416, *p* = .048). There was no significant difference in false belief ratings between language that included a medium number of mental states and none (*t*(937) = 1.587, *p* = .338).

Finally, there was a significant main effect of target paranoia on participants' mental state inference (χ^2^(1) = 6.0452, *p* = .014), such that higher paranoia was associated with a higher likelihood assigned to the true belief location; that is, participants expected that targets with higher paranoia were less likely to look for their object where they had left it.

## GENERAL DISCUSSION

Across three experiments, we investigated how cultural practices, specifically certain linguistic features of the language used to describe out‐groups in the media influence: (1) the level of empathy people feel towards out‐group members; (2) the extent to which people represent out‐group members as possessing certain personality traits, including warmth and competence; and (3) people's propensity to consider the minds of out‐group members when making inferences about their mental states.

We provide two key findings. First, we show that certain linguistic features of the language used to describe out‐group members – particularly whether that language includes mental and/or emotional states – influence the personality traits ascribed to other out‐group members. This finding held irrespective of narrative content, suggesting that the presence of mental state language influences participants' representation of out‐group minds.

The second key finding related to the tendency to take into account each out‐group member's mind when inferring their mental states, instead of assuming that all out‐group members uniformly hold a stereotypical belief. We found that participants can be more or less prone to use their representation of a target's mind to inform their inferences about the target's mental state, depending on the language used to introduce that target. Specifically, in Experiment 1 participants were more likely to use their representation of a target mind to inform their inferences about the target's mental states if that target had been introduced within an article from a left‐ relative to a right‐leaning news source. In Experiment 3, we built on this effect, showing that the presence of a high number of mental state terms increases participants' propensity to consider the minds of out‐group members when making inferences about their mental states. The fact that this effect was present only for the condition with a high number (24) of mental state terms, and not a medium (12) number, likely explains why Experiment 2 (with on average 15 mental state terms per article) did not show a strong effect of this manipulation. In these studies, we therefore demonstrate that the language used to describe out‐group members influences how people think about out‐group members' minds and that language also affects how prone people are to use their representations of their minds when making inferences about their mental states. This supports cultural evolutionary accounts of mental state inference (e.g. Heyes & Frith, 2014) which propose strong linguistic effects on the ability to infer others' mental states.

The data also provide further support for the finding that an individual's position in mind‐space (specifically, one's representation of the type of mind the individual has) is used to infer their mental states. This was first shown by Conway et al. (2020), who manipulated the portrayal of target minds such that they were represented as being more or less paranoid. Conway and colleagues showed that the representation of targets as possessing different levels of paranoia influenced the mental states ascribed to those targets, a result that we have extended here. We note that participants rarely gave the ‘true belief’ location when inferring the target's belief about the location of their object. This is consistent with Conway et al.'s findings and reflects the fact that a more paranoid target should be more likely to suspect their item has been moved, but not to know exactly where it has been moved to.

Across these studies, we examined participants' empathy for out‐group targets, as well as how competent and warm they perceived them to be, based on the language used to introduce them. In Experiment 1, we demonstrated that participants had increased empathy for targets introduced by a left‐ relative to a right‐leaning news source and also that they perceived them to be both more warm and competent. Experiments 2 and 3, however, did not find strong evidence to suggest that the presence of first‐person language or mental states was driving this effect. Besides first‐person and mental state language, there are several other features which may have differed across the articles used in Experiment 1, such as the specific topic of the article or the theme (e.g. threat to resources versus humanitarian need; Berry et al., 2016). Experiments 2 and 3 controlled for these aspects by using the same articles for each condition and altering the amount of first‐person and mental state language. It is therefore possible that some of the effects seen in Experiment 1 which did not consistently emerge in Experiments 2 and 3, particularly those relating to empathy, were the result of features such as the theme of the articles.

It is also possible that some of the variance in our results, both across and within experiments, could be due to individual differences in other factors that influence social cognitive performance, such as age, alexithymia or autism (Baron‐Cohen et al., 1985; Di Tella et al., 2020; Lecce et al., 2019). The present experiments were not designed or powered to investigate effects of such individual differences, but it should be noted that the false belief task is ‘passed’ by the majority of neurotypical children around the age of 5 years and by the majority of children with autism by the age of 9 years (see, e.g., Happé, 1995). Thus, it is unlikely that in the current neurotypical adult samples, any differences in theory of mind ability due to undiagnosed autism would impact underlying task performance. Furthermore, Experiments 1 and 3 used within‐participants designs, meaning that any inter‐individual differences could not impact our results; it is of course possible that despite random assignment of participants to condition in Experiment 2, some individual differences contributed to the weaker effects found.

Another potential limitation of the study is that, in principle, both news sources may consider immigrants to be out‐groups with respect to their readership, meaning that the differences between the sources found in Experiment 1 might not be caused solely by the two sources using different language when referring to immigrants. However, previous work (Berry et al., 2016) suggests that the left‐leaning source treats immigrants as relatively more in‐group than the right‐leaning source. It should also be noted that if our left‐leaning source were using less mental state words than might otherwise be used for in‐group targets, then if anything, Experiment 1 would be underestimating the strength of the effect of mental state language on the representation of targets' minds.

Overall, this work suggests that the media people consume impacts the way they think about others, which is consistent with cultural evolutionary accounts of how people develop the ability to understand others' minds. Given this evidence of cultural learning about out‐groups from the media, it is critical to consider how language used in different media sources shapes people's thinking about out‐group members.

These data show an effect whereby language describing out‐group members that does not include indication of their mental states, affects how readers represent the minds of those out‐group members, and the way readers make inferences about them. Specifically, when there is limited reference to mental states, people may be less likely to consider out‐group members as having individuated minds and, resultingly, different mental states, and instead attribute a stereotypical belief to all out‐group members.

Practically, if people typically use language containing fewer mental states when describing out‐group members (McClung & Reicher, 2018) and if, as is demonstrated here, recipients of that language have a reduced propensity to consider the individual minds of those described, it follows that people may consistently consider the minds of out‐group members less, relative to in‐group members, when making inferences about them. The media is one of the main ways in which people learn about others, particularly out‐groups (Banks, 2012; Esses et al., 2013; KhosraviNik, 2010; Philo et al., 2013), so the impact of cultural evolution via media language should not be understated. Indeed, exposure to a given news source may not only shape how far and how accurately a reader considers out‐group minds, but also establish and perpetuate what is considered the socially normative way of thinking about out‐groups. Whereas cultural learning theories have explained how people may acquire beliefs, knowledge and behaviours from their environment (Heyes, 2012), we here demonstrate how cultural learning may play a role in *differentially* shaping the way – and extent to which – people think about the minds of in‐group members compared to those of out‐group members.

## AUTHOR CONTRIBUTIONS

**Bryony Payne:** Conceptualization; investigation; writing – original draft; methodology; formal analysis; data curation; project administration; visualization. **Geoffrey Bird:** Conceptualization; funding acquisition; writing – review and editing. **Caroline Catmur:** Conceptualization; funding acquisition; methodology; writing – review and editing; project administration; supervision; validation.

## CONFLICT OF INTEREST STATEMENT

The authors have no known conflicts of interest to declare.

## Supporting information


Data S1.

## Data Availability

The pre‐registrations and data that support the findings of this study are openly available at: https://osf.io/6tvsd/?view_only=b4e19e9ff5e1483581abc534aea95874.

## Footnotes

^1^
This surprisingly high age was queried with Prolific who reported no reason to doubt the date of birth provided. As we did not pre‐register age as an exclusion criterion, we had no reason to exclude this participant.
