# Supplementary material for: Anti‐social learning: The impact of language on mentalizing
Source: Br J Psychol. 2025 Jul 1;116(4):1028–49. doi: 10.1111/bjop.70001 (PMC12514331; doi:10.1111/bjop.70001)
Supplement: Supplementary file 1 — Data S1. [file BJOP-116-1028-s001.docx]

**Supplemental Material: Anti-social learning: the impact of language on mentalising**

***Textual features analysis***

Given the findings of Experiment 1, we ran an exploratory textual features analysis in which we aimed to identify any linguistic factors that differed between the left- and right-leaning news sources and which could be driving these effects. Five potential variables were identified: 1) occurrences of mental state terms, 2) language valence, 3) first-person language usage, 4) readability metrics, and 5) aspects of article presentation.

These variables were subdivided for further analysis. Specifically, the occurrence of mental state terms was investigated via 6 different predictors, which separated cognitive mental states from emotional mental states. We divided the variable of ‘mental states’ into: 1a) the number of words pertaining to cognitive mental states overall; 1b) the number of words pertaining to cognitive mental states of targets (immigrants); 1c) the number of words pertaining to emotional mental states overall; 1d) the number of words pertaining to emotional mental states of targets (immigrants); 1e) the number of words pertaining to all mental states overall and, finally; 1f) the number of words pertaining to all mental states of targets.

Valence of language was investigated via: 2a) The number of positively valenced words; 2b) The number of negatively valenced words. First-person language was explored via: 3a) the number of first-person terms where the subject is a target; 3b) the number of first-person terms overall. Readability was investigated via: 4a) The Flesch Reading Ease score; 4b) the proportion of passive sentences. Both readability measures were calculated automatically using Microsoft Word. Finally, we checked aspects of article presentation, specifically: 5a) the proportion of the complete article that was included in the final task. This was because all original articles varied in length but participants were presented with only the first 350 words of each article (to the nearest end of sentence). Each article was coded for these 13 variables.

***Differences in distribution of textual features***

Wilcoxon rank-sum tests were employed to compare the distributions of these textual features between the left-leaning articles and the right-leaning articles. Results indicated significant differences in only one variable: cognitive mental state terms related to targets (p=.025), with the left-leaning articles featuring significantly more such terms compared to the right-leaning articles. Another variable, first-person terms concerning targets, approached significance with more such terms in the left-leaning articles (p=.072). Indeed, the right-leaning articles contained none of either term, raising the possibility that their absence may be related to stylistic factors. Accordingly, we opted to explore the potential effects of the number of mental states and the amount of first-person language overall, rather than confining it to language pertaining to targets.

***Impact of textual features on representation of targets’ minds***

Participant-level regressions were conducted assessing the impact of these features on the results reported in the main manuscript. Critically we were interested in whether any of these variables – and the difference between them in the two news sources – were driving participants to a) be more empathetic towards targets if those targets had been introduced by the left-leaning, relative to the right-leaning, news source and; b) be more prone to consider a target’s traits when making an inference about their behaviour.

First, we ran a regression model containing the predictor of ‘cognitive mental states’ only on empathy scores within each participant’s individual data in Experiment 1. Thereafter, we ran a t-test to assess whether the beta values arising from these regression models significantly differed from the test-statistic of zero. The t-test was not significant, suggesting that the number of cognitive mental states included in each article did not significantly predict empathy (p=.681). However, given that the original articles contained both cognitive and emotional mental states it was possible that it was the combination of cognitive and emotional states that underpinned increased empathy for targets introduced by the left-leaning news source. Thus, we ran a second regression model in each participant’s data that included the predictor of ‘all mental state words’. This t-test was significant (t(127)=3.459, p=.0007), suggesting that the number of mental states included in each article did significantly predict empathy. Finally, a Welch’s two-sample t-test was run to assess whether the beta values arising from the regression models including ‘cognitive mental states’ significantly differed to those arising from the model which included ‘all mental state words’. The t-test was significant, (t(244.76)=-2.514, p=.013), such that the mean β for ‘cognitive mental states’ was -0.042, while the mean β for ‘all mental state words’ was 0.29, suggesting that the latter predictor, which included both cognitive *and* emotional states, was a better predictor of participants’ empathy towards targets.

Given that the two news sources also neared significant differences in the amount of first-person language, we also assessed whether this variable could have an effect on empathy. The betas arising from the participant-level regression models were again entered into a one-sample t-test, which was significant (t(127)=9.495, p<.0001), suggesting that this variable also predicted participants’ empathy towards targets.

As such, in Experiment 2, we tested whether the presence vs absence of either mental/emotional state words or first-person language could explain the effects on empathy that were present in Experiment 1.

The second finding of Experiment 1 that we were interested in exploring was the linguistic feature(s) that may underpin participants’ increased propensity to consider a target’s traits when making an inference about their behaviour. Thus, the question of how target paranoia scores might interact with various linguistic features to predict the dependent variable of ‘false belief probability’ was of key interest. To explore this, we, again, ran participant-level regression models containing the predictors of ‘cognitive mental states’, ‘target paranoia scores’ as well as the interaction between the two, on false belief probability scores from Experiment 1. The betas arising from this model, tested in each participant’s data, were examined against the test statistic of 0 and the result was significant (t(127)=2.0703, p=.04045). This suggested that the number of cognitive mental states included in the articles did interact with target paranoia scores to significantly affect false belief probability ratings, suggesting that the amount of mental states within the articles did influence participants' propensity to use trait information when inferring a target’s behaviour.

To confirm whether this effect was being driven purely by cognitive mental states and not, instead, by the addition of emotional states, we again ran a model that included ‘all mental state words’ in each participant’s data. However, the subsequent t-test determined that these betas were not significantly different from zero (p=.088). As before, comparing the two sets of betas arising from the two different models (cognitive mental states only vs all mental state words) in a Welch’s two sample t-test was not-significant (p=.598), suggesting that the inclusion of emotional states – alongside cognitive mental states – was not driving the finding that people used trait information more when inferring false belief probability.

Finally, we ran regression models in participant-level data again, this time including the predictor of ‘amount of first-person language’ on false belief probability. The amount of first-person language did not interact with target paranoia scores to predict false belief probability (p=.4341).

These analyses suggested that it was only the presence of cognitive mental states within the articles that affected people’s propensity to use personality trait information to inform their inferences about a target’s behaviour. Conversely, it is the presence of more emotional states and first-person language within the articles, that led people to be more empathetic towards those targets. Thus, in Experiment 2, we aimed to manipulate the presence of both mental state (cognitive and emotional) and first-person language.
